# Potential effectiveness of an ICOPE-based long-term care intervention program for old patients with disabilities in nursing homes: protocol for a mixed methods study

**DOI:** 10.3389/fpubh.2025.1597645

**Published:** 2025-09-01

**Authors:** Rixin Qin, Kexin Huang, Zhong Li, Taiyuan Luan, Beibei Miao, Ling Gong, Wei Liu, Li Chen

**Affiliations:** ^1^School of Nursing, Beihua University, Jilin, China; ^2^School of Nursing, Jilin University, Changchun, China; ^3^The First Hospital of Jilin University, Changchun, China

**Keywords:** ICOPE, delivery of integrated healthcare, nursing homes, disability, intrinsic capacity, protocol, mixed methods intervention trial

## Abstract

**Background:**

With global population aging, functional disability has become a major public health and social care challenge. The integrated care model, centered on intrinsic capacity, aims to optimize functional abilities and improve health outcomes through systematic interventions. This approach offers innovative insights into long-term care for old patients with disabilities in institutional settings.

**Aims:**

To explore the effectiveness of an “Integrated Care for Older People (ICOPE)-Based Long-Term Care Intervention Program” compared to a control group and to assess participants’ perceptions of the program.

**Methods:**

This mixed-methods study will employ an explanatory sequential design, starting with a quantitative evaluation using a randomized controlled trial (RCT), followed by a supplementary qualitative study. The old patients with disabilities will be randomly allocated in a 1:1 ratio into the intervention group or the control group. The old patients with disabilities in the intervention group will participate in a 12-week “ICOPE-Based Long-Term Care Intervention Program,” and in the control group will maintain their routine life and standard care practices. Participant outcomes in both conditions will be assessed at pre-intervention (T_0_, week 0), post-intervention (T_1_, week 12), and 1 month after the intervention (T_2_, week 16), and a generalized linear mixed model will be used for analysis. The primary outcome is the change in intrinsic capacity, with the significance of the mean difference assessed to determine the intervention effect. In the qualitative part of this study, interviews will be conducted with old patients with disabilities from the intervention group at T_1_ to explore their experiences of receiving the intervention, and content analysis will be applied to the data collected.

**Discussion:**

This study will assess the effectiveness of an “ICOPE-Based Long-Term Care Intervention Program” for old patients with disabilities in nursing homes. If effective, it could provide a feasible and structured approach to improving long-term care quality in institutional settings.

**Trial registration:**

Identifier, ChiCTR2400094580.

## Highlights

- A mixed-methods study to enhance the intrinsic capacity of old patients with disabilities in nursing homes through an ICOPE-based long-term care intervention program.
- Study population will include old patients with disabilities in nursing homes.
- The outcomes of the ICOPE-based long-term care intervention program will be evaluated using a comprehensive and detailed assessment based on the ICOPE intrinsic capacity screening tool proposed by the World Health Organization (WHO). This includes assessments of cognitive function, motor function, sensory function (vision and hearing), vitality, and psychological function.
- Blinding of participants and trainers will not be possible, increasing the risk of bias.

## 1 Introduction

With the further acceleration of global aging, the number of old patients with disabilities is increasing rapidly (1). The old patients with disabilities are those whose daily living abilities are partially or completely lost for a prolonged period (over 6 months) due to physiological aging, pathological damage, or physical and mental disabilities (2). United Nations statistics indicate that over 46% of the global population aged 60 and above live with disabilities, and more than 250 million older adults endure moderate to severe disabilities (3). According to projections, the number of older adults with disabilities in China is expected to continue rising steadily. By 2030, the proportion of old people with disabilities is projected to exceed 57%, reaching more than 77.66 million (4). In the absence of effective prevention and control strategies, this figure is anticipated to surpass 70% by 2050 (4). In this context, nursing homes, as the primary living and care setting for old patients with disabilities, warrant particular attention regarding the issue of disability (5).

Despite the significant advantages nursing homes show in providing professional care services (6), they still face some pressing challenges. First, the fragmentation of services is a prominent issue (7). The health problems of older adults are complex and long-term, with chronic diseases and geriatric syndromes being common (8). Without coordinated interventions, these issues may lead to polypharmacy, hospitalization, or even death. Secondly, the care service model is singular. Long-term care services mainly focus on physical care and functional rehabilitation for old patients with disabilities (9), lacking a comprehensive consideration of their multidimensional health needs, such as in exercise, cognition, nutrition, and other aspects. This limitation not only affects the effectiveness of care but also hinders the overall health recovery of older adults (10), placing enormous pressure on the healthcare system and social care services. Therefore, it is crucial to explore comprehensive and integrated long-term care strategies.

In its Global Report on Aging and Health (11), the WHO introduced two key concepts: intrinsic capacity and functional ability. Intrinsic capacity refers to the overall physical and psychological abilities of an individual (12), while functional ability refers to the combination and interaction of an individual’s intrinsic capacity with their environment, reflecting their ability to perform various functions (13). A shift in older adult care involves moving from focusing solely on treating a specific disease or health issue to maximizing the intrinsic capacity of older adults throughout their life course (14).

The decline in intrinsic capacity is a key predictor of disability, with a significant correlation between the two (15–18). Numerous studies conducted in communities, nursing homes, and hospitals have shown that the decline in one or more dimensions of intrinsic capacity is closely associated with disability (15–18). The WHO has also emphasized the identification of conditions associated with the loss of intrinsic capacity, providing opportunities for interventions to slow, halt, or reverse the downward trend (19). Therefore, early assessment and intervention in the decline of intrinsic capacity are of great significance for optimizing long-term care services for old patients with disabilities.

Building on the WHO’s Global Report on Aging and Health (11), the WHO introduced the ICOPE framework in 2017 (14), which focuses on optimizing the functionality and intrinsic capacity of older adults to enhance their overall health. As the WHO states, the complexity of health issues in older adults requires health services to be provided in a more integrated manner, with the goal of maximizing or improving the intrinsic capacity of older adults and promoting their functional abilities (20). To operationalize this goal, the “ICOPE Handbook: Guidance for Person-Centered Assessment and Pathways in Primary Care” outlines a five-step care pathway for the assessment and management of declines in intrinsic capacity among older people: (1) Screening for possible declines in intrinsic capacity; (2) Comprehensive assessment of health conditions related to the decline in intrinsic capacity; (3) Development of a personalized care plan; (4) Ensuring referral pathways and monitoring of the care plan; (5) Community engagement and caregiver support. This systematic process provides a structured foundation for delivering integrated care in community.

Currently, practice-based projects centered around intrinsic capacity, at the heart of the ICOPE framework, are being gradually launched to improve the quality of care for older adults. These ICOPE practice projects have been implemented in multiple countries worldwide, including France (21) and South Korea (22), achieving significant results. In China, researchers such as Ma et al. (23) conducted a study on 976 middle-aged and older adults (aged 50–97) enrolled in the ICOPE-China project, using the ICOPE screening tool for intrinsic capacity assessments. The study found that 69.1% of participants showed a decline in intrinsic capacity, validating the tool’s effectiveness in identifying declines in the intrinsic capacity of older adults and providing valuable insights for the further development of older adult care in China. Overall, while domestic projects primarily focus on the initial screening of intrinsic capacity, there is still a lack of systematic research and practical application regarding the design and implementation of comprehensive care pathways and intervention programs, particularly for old patients with disabilities in nursing homes.

The main aim of this study is to explore the effects and the experiences of using “long-term care intervention program based on ICOPE for old patients with disabilities in nursing homes” in old patients with disabilities. An explanatory sequential design will be used, starting with a RCT investigating the impact of a long-term care intervention program on intrinsic ability (motor function, cognitive function, psychological function, sensory function, and nutrition) in a group of old patients with disabilities. Subsequently, qualitative surveys will be conducted to gain an in-depth understanding of participants’ subjective experiences of participating in this exergame intervention, in particular the benefits arising from the intervention as well as the influencing factors for participation, which will help understanding the mechanisms behind the therapeutic effects. This information will provide a basis for optimizing, refining, and promoting long-term care intervention program.

We hypothesis that participants in the long-term care intervention program group will experience an improvement of intrinsic ability, compared to those in the control group. Furthermore, we anticipate that these improvements will be maintained at 1-months follow-up.

## 2 Methods

### 2.1 Study design

**Figure 1:**
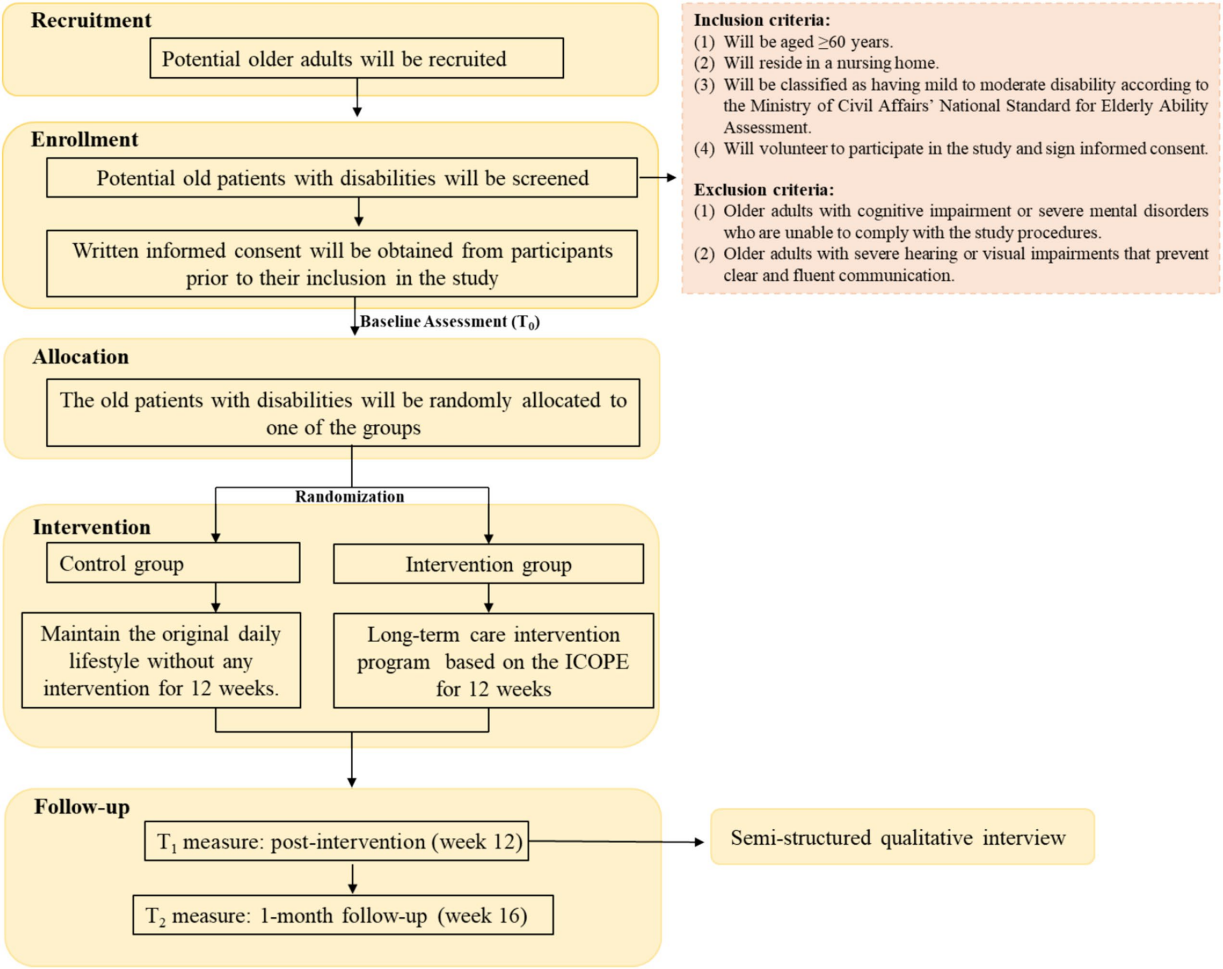
Study design flowchart.

This mixed-methods study will consist of an assessor-blinded, parallel-group RCT and a qualitative component. The design will align with the Medical Research Council’s guidance for the evaluation of complex interventions (24). An explanatory sequential design will be adopted, beginning with a quantitative, outcome-based assessment of treatment effects, followed by a qualitative investigation to explore participants’ perceptions of the intervention. The qualitative study will allow understanding the real experiences of old patients with disabilities in nursing homes participating in the intervention and the mechanisms behind the treatment (25). The study design is illustrated in Figure 1.

The study will be prepared in accordance with the Good Reporting of a Mixed Methods Study (GRAMMS) guidelines (26) and the Standard Protocol Items: Recommendations for Interventional Trials (SPIRIT) statements (27, 28). This trial was prospectively registered at Chinese Clinical Trial Registry (number: ChiCTR2400094580) on 25nd December 2024.

### 2.2 Sample size and power calculations

In quantitative research, this study will use a completely randomized design for sample size estimation. A repeated measures ANOVA *F*-test in G*Power 3.1 will assess the main effect of the within-between interaction. Parameters will be set as follows: effect size = 0.25, *α* = 0.05, 1-*β* = 0.90. The correlation among the three repeated measures was set to *r* = 0. Note that this reflects a conservative approximation and any correlation larger than *r* = 0 would have reduced the required amounts of participants. Software calculations will determine a required 70 participants (35 per group). Accounting for a 20% attrition rate, the final sample size will be 42 per group, totaling 84 participants.

In qualitative research, purposive sampling will be used to select old patients with disabilities in nursing homes who have completed the 12-week intervention. Recruitment, interviews, and data analysis will be conducted sequentially in an iterative process until data saturation is reached, determining the final sample size (29).

### 2.3 Eligibility and recruitment

Participants will be recruited from nursing home in Changchun, Jilin Province, China. The old patients with disabilities in nursing homes will be recruited through live lecture, poster promotion and official account by researchers and staff. Eligible subjects will be screened through face-to-face surveys. Inclusion criteria for participants will be: (1) Will be aged ≥60 years; (2) Will reside in a nursing home; (3) Will be classified as having mild to moderate disability according to the Ministry of Civil Affairs’ National Standard for Elderly Ability Assessment; (4) Will volunteer to participate in the study and sign informed consent. Participants will be excluded if: (1) Older adults with severe cognitive impairment or severe mental disorders who are unable to comply with the study procedures; (2) Older adults with severe hearing or visual impairments that prevent clear and fluent communication.

### 2.4 Randomization, allocation and blinding

Participants will be randomly allocated to the intervention or control groups in a 1:1 ratio using computer-based permuted block randomization. The randomization sequence will be generated by an independent research coordinator, and the details of group allocation will be concealed on cards placed inside sequentially numbered, sealed opaque envelopes. Outcome evaluators, and data analysts will be kept blinded.

### 2.5 Interventions

#### 2.5.1 Development of the intervention program

A research team (a total of five members) comprising one professor of nursing, one associate professor of nursing, two doctoral students, and one master’s student will be established to develop the initial framework of the intervention program. Guided by the ICOPE model, the study will focus on the management and care pathways for the decline in intrinsic capacity, incorporating findings from previous studies and guideline recommendations. The intervention program, designed for the long-term care of old patients with disabilities in nursing homes, will adopt a multi-dimensional approach to address the decline in intrinsic capacity. It will encompass motor function, cognitive ability, vitality, sensory perception (vision and hearing), psychological function, and health education to enhance the overall health and quality of life of old patients with disabilities.
To ensure the scientific validity and feasibility of the intervention program, this study will adopt the Modified Delphi Method to refine and finalize the proposal (30, 31). The specific steps are as follows: (1) A research team will be established. (2) Based on prior research and in-depth discussions, the team will develop an expert consultation questionnaire comprising three key components: ① A situation description module, which will outline the purpose of the consultation, the research background, and the theoretical framework; ② An importance assessment module, where experts will evaluate the significance of various elements of the intervention program; ③ An expert authority assessment module, which will gather information on each expert’s background, professional experience, and familiarity with the research topic. (3) The research team will select 15–50 experts from relevant fields to ensure the authority and representativeness of the consultation process (32). (4) The first round of expert consultation will be conducted, with responses undergoing preliminary analysis and the questionnaire revised accordingly based on expert feedback. (5) A second round of expert consultation will then be carried out. If the level of consensus among experts does not meet the predefined threshold, additional rounds will be conducted as needed. If sufficient consensus is reached, the consultation process will be concluded. Statistical analysis will be performed on the final consultation results, and the intervention program will be revised accordingly based on expert feedback. Once a stable consensus is reached, the research team will finalize the intervention program (Table 1), ensuring its scientific rigor, feasibility, and clinical applicability.

**Table 1:** ICOPE-based long-term care intervention program for old patients with disabilities in nursing homes.

Main domain	Intervention indicators	Intervention content	
1. Motor function	1.1 Aerobic exercise	1.1.1 Walking 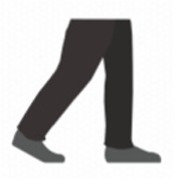	(1) Walk facing forward: Keep your gaze straight ahead and avoid looking down at the ground. When stepping forward, first firmly place your heel on the ground, followed by the toes. Keep your shoulders relaxed and allow your arms to swing naturally to help maintain balance, ensuring a stable and safe walking posture. (2) Each walking session consists of 5 sets, with each set lasting 2–5 min. After completing one set, take a 1-min rest between sets to adjust your posture and relieve physical fatigue before proceeding to the next set. This cycle continues until all 5 sets are completed.
	1.2 Resistance training	1.2.1 Lifting a water bottle 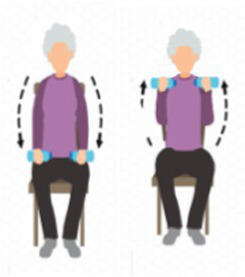	(1) Seated Position and Movement: While performing the exercise in a seated position, ensure that both hands hang naturally at your sides, each firmly holding a water bottle. Slowly bend your elbows toward your chest while engaging your arm muscles, lifting the bottles smoothly until they reach shoulder height. Hold this position briefly to feel the contraction and extension of the arm muscles. (2) Repetitions and Rest: Each training session consists of 3 sets of 12 repetitions. After completing one set, take a 1-min rest before proceeding to the next set. • Progressive Intensity: After six weeks of continuous training, to further enhance the effectiveness of the exercise, the amount of water in the bottles can be gradually increased to intensify the workout. It is essential to increase the water level progressively, avoiding excessive increments at once to ensure a safe and steady progression in training.
		1.2.2 Squeezing a ball 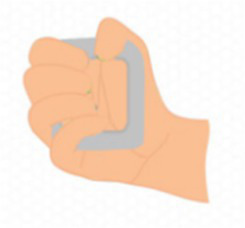	(1) Hand Exercise Technique: During the exercise, gradually apply pressure to squeeze the ball at a slow and controlled pace, feeling the tension and contraction in the hand muscles. Once reaching maximum grip strength, hold the position briefly. Then, slowly release the grip, allowing the hand muscles to relax and stretch. Repeat the squeezing and releasing motion to complete one set. (2) Repetitions and Rest: Each session consists of 3 sets of 12 repetitions (squeeze and release). After completing one set, take a 1-min rest before proceeding to the next. Once all 3 sets are completed with one hand, switch to the other hand and repeat the exercise following the same steps to ensure balanced hand muscle strengthening.
	1.3 Balance training	1.3.1 Simulated sitting motion 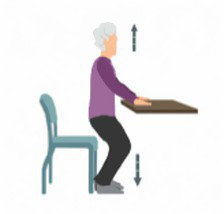	(1) Exercise Technique: Stand in front of a table and position yourself securely. Slowly bend your knees and lower your hips as if you are about to sit down, maintaining a smooth and controlled motion throughout. Once you reach the desired squat depth, gradually engage your muscles to return to the initial standing position. To ensure safety during the exercise, it is recommended to place a chair behind you for support if needed. (2) Repetitions and Rest: Each session consists of 3 sets of 12 repetitions. After completing one set, take a 1-min rest before proceeding to the next set.
		1.3.2 Walking on tip toes and heels 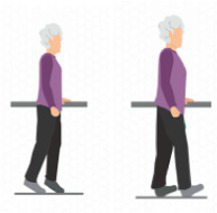	(1) Exercise Technique: Stand up steadily and hold onto the edge of a table or a nearby handrail for support. Shift your body weight onto your toes, using them as the primary support, and take seven controlled steps forward while maintaining balance. After completing the toe-walking sequence, take a brief rest to adjust. Then, shift your weight onto your heels, using them as the main support, and walk at least seven steps forward. This exercise effectively strengthens leg muscles and improves balance. (2) Repetitions and Rest: Each session consists of 3 sets of 12 repetitions. After completing one set, take a 1-min rest before proceeding to the next.
	1.4 Flexibility training	1.4.1 Seated arm stretch 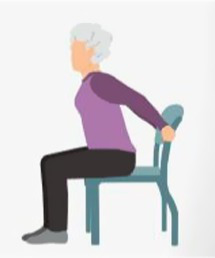	(1) Exercise Technique: Sit securely on a chair, ensuring a slight gap between your back and the chair’s backrest. Let your arms hang naturally at your sides, then slowly extend them backward, attempting to grasp the back of the chair. Once you successfully hold the chair, maintain this position while pushing your chest forward until you feel a slight stretch in your arm muscles. Hold this posture for 10 s, then relax for 5 s while keeping your hands on the chair. (2) Repetitions and Rest: Each session consists of 3 sets of 3 repetitions. After completing one set, take a 30-s rest before proceeding to the next set.
		1.4.2 Overhead arm stretch 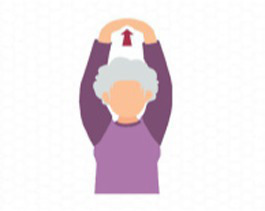	(1) Exercise Technique: This exercise can be performed in either a standing or seated position. Once ready, interlock your fingers and extend your arms straight upward, as if reaching for the ceiling. Hold this stretch for 10 to 12 s, fully feeling the extension in your arm muscles. After each repetition, take a brief pause and relax your arms for 5 s to allow the muscles to recover before repeating the movement as needed. (2) Repetitions and Rest: Each session consists of 3 sets of 3 repetitions. After completing one set, take a 30-s rest before proceeding to the next set.
2. Vitality	2.1 Balanced diet	2.1.1 Dietary recommendations	(1) Adjusting Meal Plans: Based on the nutritional status of disabled older adults, nutritionists are advised to modify dietary plans accordingly to meet their specific needs. (2) Establishing Healthy Eating Habits: Older adults often have insufficient food intake. To encourage greater food consumption, it is recommended that they dine in groups, fostering a more enjoyable and social eating experience.
		2.1.2 Oral nutritional Supplementation	(1) Oral Nutritional Supplementation: Additional high-quality protein, energy (carbohydrates), and essential micronutrients (such as vitamins and minerals) can be provided based on individual needs, preferences, and physical conditions. (2) Protein Supplementation: As protein absorption declines with age, it is recommended that older adults increase their protein intake to maintain muscle strength and overall health.
	2.2 Multimodal exercise	Refer to 1. Motor function	
	2.3 Weight management	Regular weight monitoring	Closely monitor body weight once per week.
3. Psychological function	3.1 Psychological interventions	3.1.1 Cognitive behavioral therapy	(1) Identifying problems in daily life. (2) Becoming aware of thoughts, emotions, and beliefs related to these problems. (3) Recognizing negative or inaccurate thoughts. (4) Restructuring these thoughts to make them more realistic.
		3.1.2 Problem-solving counseling	For disabled older adults experiencing depressive symptoms or some degree of social dysfunction (without a diagnosed depressive episode or disorder), biweekly counseling sessions will be conducted. These sessions aim to identify psychological issues in a timely manner and provide support and guidance as needed.
		3.1.3 Behavioral activation	Behavioral activation involves encouraging disabled older adults to participate in rewarding activities to help alleviate depressive symptoms.
		3.1.4 Life Review Therapy	Digital reminiscence intervention system, through the mobile (mobile phone/tablet) clicking on the module as well as voice interaction, to complete the construction of reminiscence themes and reminiscence resource library and other digital reminiscence content based on subthreshold depressed older adults. In total, it includes a number of modules such as personal information, assessment module, age footprint, chronological impression, point reward, and settings.
	3.2 Multimodal exercise	Refer to 1. Motor function	
	3.3 Mindfulness practice	3.3.1 Mindful movement	There are various types of mindfulness practices, with one of the most commonly used methods being sitting or lying quietly while focusing attention on the sensation of breathing. Additionally, mindful movement, such as practicing mindfulness while walking, can be beneficial for some individuals.
4. Cognitive ability	4.1 Cognitive stimulation	4.1.1 Computer-assisted cognitive system	With the help of mouse and keyboard operation, the computerized cognitive aid system (virtual supermarket training system) can complete the preset task modules of the system in order to experience virtual shopping in an immersive way, and then achieve the goal of cognitive training in multiple fields. The system covers nine task modules, each corresponding to a precise cognitive training, specifically: ‘Memory list’ to train intentional memory; ‘Map navigation’ to exercise the ability to navigate; ‘Voice broadcasting Each module precisely corresponds to a cognitive training task, as follows: ‘Memory list’ to train intentional memory; ‘Map navigation’ to improve navigation skills; ‘Voice announcement’ to enhance unintentional memory; ‘Labeling’ to strengthen verbal naming; ‘Voice cashier prompts’ to enhance verbal receptivity; ‘Payment calculation’ to train ‘Payment Calculator’ trains numeracy; “Date Code” fosters orientation; “Prize Redemption” exercises sustained attention; and “Bus Decision Making” enhances decision-making skills. By participating in these tasks, users can train their cognitive abilities in multiple domains, such as memory, language function, executive function, attention and time orientation.
	4.2 Multi-modal exercise	Refer to 1. Motor function	
5. Sensory	5.1 Hearing devices	5.1.1 Hearing aids	Hearing aids are often the most effective solution for age-related hearing loss. They amplify sound and are beneficial for most individuals. Hearing aids can be worn over or inside the ear, making them convenient to use. However, it is essential to explain to older adults that hearing aids do not cure or reverse hearing loss, but rather help improve auditory perception.
		5.1.2 Cochlear Implants	Cochlear implants are surgically placed inside the ear to convert sound into electrical impulses, which are then transmitted to the auditory nerve. A thorough evaluation is necessary to determine whether a cochlear implant is suitable. If implantation is not possible or appropriate, older adults and their families should be provided with information and training on lip reading and sign language as alternative communication methods.
		5.1.3 Audio induction loops and personal sound amplifiers	Audio induction loops and personal sound amplifiers are also effective. An audio induction loop (or hearing loop) consists of one or more wires placed around a designated space, such as a conference room or service counter. These wires transmit sound signals from a microphone and amplifier directly to compatible hearing aids, improving auditory clarity in specific environments.
		5.1.4 Social and natural environment	(1) Regular social interaction can help reduce the risk of cognitive decline, depression, and other emotional and behavioral consequences of hearing loss. (2) Social support networks can provide assistance during particularly challenging situations. (3) Peers and caregivers play a crucial role in preventing loneliness and social isolation. They may need guidance on how to facilitate social engagement, such as maintaining communication with individuals with hearing loss and organizing activities that encourage their participation in social networks.
	5.2 Communication strategies for caregivers		(1) Ensure your face is visible when speaking to the individual. (2) Maintain adequate lighting on your face to help the listener see your lips clearly. (3) Gain the attention of the person with hearing loss before speaking. (4) Minimize distractions, particularly loud noises and background sounds. (5) Speak clearly and at a moderate pace—do not shout. (6) Do not avoid communicating with individuals with hearing loss, as social isolation can lead to depression and emotional distress.
	5.3 Vision rehabilitation	5.3.1 Magnifiers	Magnifiers can often provide assistance for individuals with vision impairment. Simple magnifiers are affordable and come in various magnification levels. However, they only enlarge nearby objects. If a basic magnifier does not adequately address the issue, a comprehensive eye examination is recommended.
		5.3.2 Irreversible vision loss	Many individuals experience low vision that cannot be fully corrected with prescription glasses. For these individuals, assistive visual devices such as desktop or portable magnifiers offer higher magnification than regular eyeglasses. These devices can enhance near-vision tasks, including reading books or newspapers, identifying currency, reading labels, and examining small objects or specific parts of larger objects.
	5.4 Adapting to low vision	5.4.1 Improving lighting	Good lighting is especially important for near vision. Ideally, light should come from the side to prevent shadows from interfering with visibility.
		5.4.2 Reducing glare	Bright lighting is generally beneficial; however, sunlight or intense artificial light may cause discomfort for some individuals. Adjusting brightness or using diffused lighting can help reduce glare.
		5.4.3 Removing obstacles	Hazards such as furniture and other hard objects should be removed from the daily paths of individuals with low vision. If removal is not possible, these objects should always remain in a fixed location to prevent accidents.
		5.4.4 Enhancing contrast	Good contrast between and within objects makes them easier to see, locate, or avoid. Examples include high-contrast markings on stair edges (especially for individuals with vision in only one eye), using colored plates to make food more visible, and writing with black pens for better readability. Individuals with low vision, their family members, and caregivers can enhance the visibility and safety of household and kitchen tools by adding color-coded handles. For instance, wrapping brightly colored tape around knife handles or painting them can help improve clarity and safety.
6. Health education and guidance	6.1 Prevention guidance for disability and related diseases	Thematic education sessions will be held once per week, covering a range of topics including disability care management, long-term care management, exercise guidance, cognitive decline management, social interaction guidance, psychological well-being support, balanced diet recommendations, sleep guidance, proper medication use, fall prevention, assistive device usage, and disease prevention and treatment education.	
6.2 Medication guidance			
6.3 Sleep and rest			
6.4 Use of assistive devices			

#### 2.5.2 Pre-experiment

A one-week pilot study will be conducted using the preliminary intervention program with 10 older adults with mild to moderate disabilities in a nursing home in Changchun to assess their feedback and refine the design and implementation strategy of the intervention plan. The results are expected to indicate that, during the initial phase of the intervention, participants will generally be unfamiliar with the intervention measures and may exhibit a certain level of apprehension, highlighting the need for a gradual process of familiarization and adjustment. Additionally, nursing home staff are likely to report that they have not previously been exposed to a systematic long-term care intervention program, thereby underscoring the necessity for targeted training on intervention procedures and key implementation considerations.
Based on these findings, the research team will revise the intervention program accordingly. Modifications will include strengthening pre-intervention training sessions for nursing home staff to facilitate smooth and consistent implementation. During the intervention period, staff will be encouraged to offer individualized assistance tailored to the needs of each older adult, with a gradual shift toward independent participation as participants become more comfortable with the content, ensuring both safety and active engagement. Regarding the psychological well-being component, in light of staff feedback, the originally planned weekly problem-solving consultation or therapy sessions will be adjusted to a biweekly schedule. Similarly, the frequency of the reward system will be modified from once per week to once every 2 weeks, in order to balance participant engagement with the practical feasibility of program delivery.

#### 2.5.3 Formal intervention

##### 2.5.3.1 Intervention group

Before the intervention, two preparatory practice sessions will be conducted over a 2-week period. During the first week, a trained researcher will introduce and demonstrate the equipment operation procedures, guiding participants through the exercises to help them become familiar with the system’s functions and usage methods. In the second week, two researchers will evaluate each participant’s operational proficiency and provide individualized instruction as needed, ensuring that all participants develop a clear and accurate understanding of how to use the system independently. In addition, at the conclusion of the training, participants will be given a video tutorial recorded by the research team, demonstrating the operation procedures and key methods. This resource will support those who may require additional time and reinforcement to gain confidence with the system.

The intervention group will participate in the study for a total duration of 16 weeks, comprising a 12-week intervention phase followed by a 4-week follow-up phase. The old patients with disabilities in intervention group will take part in the “ICOPE-Based Long-Term Care Intervention Program for old patients with disabilities in Nursing Homes” (Table 1), in addition to receiving the same routine care as the control group. The intervention program was designed to enhance physical, nutritional, psychological, cognitive, and sensory functions, as well as to provide comprehensive health education and guidance. It comprised the following six components: (1) Motor function: training incorporated resistance training, aerobic exercise, flexibility training, and balance training, with each session lasting 30–45 min, twice per week. (2) Nutrition: nutritional support was tailored to individual needs based on nutritional status assessments, ensuring a balanced diet and appropriate oral supplementation. (3) Psychological function: ① Problem-solving counseling or therapy, 30–45 min per session, once every 2 weeks; ② Behavioral activation with a reward system, 30–45 min per session, once every 2 weeks. (4) Cognitive function: computer-assisted cognitive training using the virtual supermarket training system, 30–45 min per session, twice per week. (5) Sensory function: ① Auditory training using cards, 30–45 min per session, once weekly; ② Visual training using a bouncy ball, 30–45 min per session, once weekly. (6) Health education and guidance: it was delivered through thematic education sessions, covering essential health topics, conducted once per week for 30–45 min. All intervention sessions will be conducted in the nursing home’s activity room to ensure accessibility and consistency for participants.

##### 2.5.3.2 Control group

The old patients with disabilities assigned to the control group will continue their routine daily activities and receive standard care provided by the nursing home, without exposure to any components of the intervention program. During the intervention period, researchers will conduct non-intrusive observation and data collection, refraining from making any active changes to participants’ dietary patterns, daily routines, or recreational activities.

### 2.6 Outcome measures

#### 2.6.1 Quantitative outcome measures

The participants in both the intervention and control groups will be assessed on their intrinsic capacity, covering six domains, including cognitive function, motor function, vitality, vision, hearing, and depression. Assessments will be conducted at T_0_, T_1_, and T_2_. For a detailed description of data collection, refer to Table 2.

**Table 2:** Overview of data collection.

Measures	T_0_	T_1_	T_2_
Demographics	△		
ICOPE screening tool	△	△	△
MMSE	△	△	△
SPPB	△	△	△
MNA	△	△	△
Visual acuity test	△	△	△
Hearing test	△	△	△
PHQ-9	△	△	△

T_0_, pre-intervention (week 0); T_1_, post-intervention (week 12); T_2_,1-month follow-up (week 16); ICOPE, Integrated Care for Older People; MMSE, Mini-Mental State Examination; SPPB, Short Physical Performance Battery; MNA, Mini Nutritional Assessment; PHQ-9, Patient Health Questionnaire-9.

##### 2.6.1.1 ICOPE intrinsic capacity screening tool

The assessment of intrinsic capacity strictly follows the five domains proposed by the WHO (33): cognition function, motor function, sensory function (Vision and Hearing), vitality, and psychological function. Each domain is scored as 0 (normal) or 1 (impaired), and the total intrinsic capacity score is the sum of the five domain scores, ranging from 0 to 5. A score of 3 to 5 is defined as low intrinsic capacity, while a score of 0 to 2 is classified as high intrinsic capacity. Specific contents include: ① Cognitive function: Screening assesses memory and temporal orientation, including word recall (flower, door, rice), time and place orientation, and short-term memory tests. An incorrect response or failure to recall all three words indicates a positive screening result. ② Motor ability: The chair rise test is used to assess mobility. Participants must stand up and sit down five times consecutively without using their arms for support, completing the task within 14 s. Failure to meet this requirement is considered a positive screening result. ③ Nutrition: Assesses weight changes and dietary habits over the past 3 months, including whether the individual has experienced weight loss of ≥3 kg or frequently finds food tasteless or difficult to swallow. A “yes” to either question indicates a positive screening result. ④ Vision: Assess whether there are visual difficulties (e.g., watching TV, reading, or recognizing faces) or a history of eye disease treatment. A positive response to either indicates a positive screening result. ⑤ Hearing: Use the whisper test or inquire about subjective hearing perception. Failure in the test or a significant perceived decline in hearing is considered a positive screening result. ⑥ Depression: Assess recent emotional state, including feelings of low mood, depression, or hopelessness, or loss of interest in daily activities. The presence of these symptoms indicates a positive screening result.

##### 2.6.1.2 Mini-mental state examination

The MMSE was developed by Folstein et al. (34) as a cognitive screening tool. It consists of 30 items covering orientation (time/place), memory, attention, calculation, language, and visuospatial abilities, with a total score of 30 points. A score of 24 or above is considered indicative of normal cognitive function, with higher scores reflecting better cognition. The MMSE is easy to administer, has clearly defined scoring criteria, and takes only 5–10 min to complete. It demonstrates good sensitivity, specificity, and reliability, making it one of the most widely used cognitive screening tools worldwide. Research has shown that MMSE scores are influenced by educational attainment (35). Therefore, education-stratified cut-off values have been recommended: ≤19 for illiterate individuals, ≤22 for those with primary education (≤6 years), and ≤26 for those with junior high school education or above.

##### 2.6.1.3 Short physical performance battery

The SPPB scale will be used to assess the motor function of the study participants (36). The SPPB consists of three components: balance, gait speed, and lower limb strength, which are assessed through three tests: balance test (standing with feet in side-by-side, semi-tandem, and tandem positions), 4-meter walking speed test (measuring the time taken to walk a 4-meter distance), and the repeated chair stand test (5 repetitions of standing up from a chair). The total score ranges from 0 to 12, with higher scores indicating better physical function and lower levels of activity limitations. According to the established scoring criteria, a score of ≥10 indicates normal motor function, while a score of <10 indicates abnormal motor function.

##### 2.6.1.4 Mini nutritional assessment

This study will use the MNA scale (37) to assess the nutritional status of the participants. The MNA scale consists of 4 sections and 18 questions, covering anthropometric measurements (e.g., weight, height, arm circumference), overall assessment (lifestyle, activity level), dietary evaluation (types and amounts of food, eating habits), and subjective assessment (self-rating and others’ evaluations). The assessment process is as follows: If the score for the first 6 items is >12, it indicates normal nutritional status, and no further evaluation is needed. If the score is ≤12, it suggests the risk of malnutrition, and further evaluation is required. The total score ranges from 0 to 30, with ≥24 indicating good nutrition, 17–24 suggesting risk of malnutrition, and <17 indicating malnutrition (37).

##### 2.6.1.5 Visual acuity test

This study will follow the WHO-recommended visual acuity test, including both distance and near vision assessments. Distance vision measurement: The participant will be asked to remove their glasses and stand 3 meters away from the visual acuity chart. The left, right, and both eyes will be tested. If the unaided distance vision of one eye is below 4.5, it will be considered impaired. Near vision measurement: The participant will be asked to remove their glasses and stand 33 cm away from the near vision chart. The left, right, and both eyes will be tested. If the unaided near vision of one eye is below 1.0, it will be considered impaired. Comprehensive visual function assessment: If any of the distance or near vision results for the left, right, or both eyes show impairment, the participant’s vision will be considered abnormal. Otherwise, if all vision test results are normal, the participant’s vision will be considered normal.

##### 2.6.1.6 Hearing test

The hearing test will be conducted using an automated application to ensure accuracy and reliability. The testing environment will require ambient noise to be <40 dB. The “Hearing Bao” APP will be used to test both the left and right ears, and ambient noise levels will be recorded. The test will include frequencies of 125 Hz, 250 Hz, 500 Hz, 1,000 Hz, 2000 Hz, 4,000 Hz, and 8,000 Hz. However, for analysis, only the data from the WHO-recommended frequencies of 500 Hz, 1,000 Hz, 2000 Hz, and 4,000 Hz will be used. According to the standards, if the hearing test result for any ear in these four frequencies is ≤35 dB, the hearing function for that ear will be considered normal. Hearing function will be considered normal only when the test results for both ears are normal across all four frequencies.

##### 2.6.1.7 Patient health Questionnaire-9

The PHQ-9 (38) contains nine items, including loss of pleasure, low mood, sleep difficulties, lack of energy, eating disorders, low self-esteem, concentration difficulties, slow movement, and self-harm/suicidal ideas. These items use a 4-point Likert scale, ranging from “not at all” to “nearly every day.” The total score ranges from 0 to 7, with a higher score indicating a higher severity. This scale had a satisfactory Cronbach’s *α* coefficient among students (39) and the internal consistency of the scale was also good.

##### 2.6.1.8 Adherence, adverse events, and satisfaction

In the empirical study of long-term care intervention program based on ICOPE for old patients with disabilities in nursing homes, adherence, adverse events, and satisfaction are key indicators for evaluating the intervention’s effectiveness. Adherence reflects the degree to which participants follow the prescribed intervention, including the frequency and duration of their participation. Adverse events are any untoward medical occurrences in a participant during a clinical trial. To ensure participant safety, a response protocol will be implemented. If a participant becomes distressed or unwell and cannot attend a session, it will be rescheduled based on their condition. Participants will be closely monitored by nursing home staff and the research team, with timely medical support provided as needed. The intervention will be paused if continued participation poses a risk, and clinical assessment will be arranged if necessary. All adverse events will be documented and reported to the ethics committee. Satisfaction will be assessed to capture participants’ subjective experiences, perceived benefits, and attitudes toward the intervention, which is critical for evaluating the intervention program’s acceptability and for informing future improvements.

#### 2.6.2 Qualitative data collection

The old patients with disabilities in nursing homes who have completed the 12-week “long-term care intervention program based on ICOPE for old patients with disabilities in nursing homes” will be invited to participate in focus group interviews to explore their experiences of taking part in the intervention. Table 3 shows the details of the interview outline.

**Table 3:** Semi-structured interview outline.

Number	Questions
1	What were the main reasons you initially enrolled in this program? Please explain and rank them by importance.
2	How did you feel and what were your experiences after participating in this care intervention program?
3	What challenges did you encounter during the implementation of this care intervention program? What was the biggest challenge? How was it resolved? Please provide an example.
4	Would you be willing to continue participating in this care program in the future? Would you recommend this program to others around you? Please explain.
5	How do you think this care intervention program benefits you compared to traditional care models?
6	What kind of help and support would you like to receive in the future at the nursing home? Are there any areas that need improvement or further development? Please explain.
7	Is there anything else you would like to add to today’s conversation?

### 2.7 Quality control

Our team is an experienced group of researchers, consisting of senior experts and graduate students specializing in nursing. If there are complex clinical issues, the researchers in our team and professional experts will work together to find solutions. The researchers will deliver consistent and standardized training to all personnel involved in implementing the intervention. This group-based training will include demonstrations and assessments of scale usage to ensure that implementers are well-versed in both the content and application of the scales. Additionally, on-the-spot testing and questionnaire response collection will be carried out to guarantee the validity and accurate recall of the questionnaire data.

### 2.8 Statistical analysis

For quantitative analysis, Epidata 3.1 (Epidata Association) will be used for double data entry and SPSS 26.0 (International Business Machines Corporation, IBM) will be used for data analysis and processing. Descriptive statistics will be used to describe participants’ characteristics and mental health outcomes. For continuous variables, the Shapiro–Wilk test (S-W test) will be used to assess normality. Depending on the results of the S-W test, the mean and standard deviation or the median and the interquartile range will be used for descriptive statistics. For categorical variables, frequencies and percentages will be reported. Independent samples *t*-tests, Mann–Whitney U tests and χ^2^ tests will be used to compare outcome results and baseline data. Paired sample t-tests or non-parametric rank sum tests will be used to compare the outcomes within the group. To analyze between-group differences, a two-independent sample t-test or a Mann–Whitney U rank sum test will be used. The changing trend of the outcome indicators of the study subjects will be measured between T_0_, T_1_, and T_2_; the inter-group factor will be the subgroup (intervention group and control group), the intra-group factor will be the measurement time point (T_0_, T_1_, and T_2_), and the interaction will be the group × time point. A generalized linear mixed model will be used for analysis. In addition, age, frailty status, and other relevant variables will be included as covariates, depending on data availability and implementation feasibility, to control for their potential confounding effects. The significance level of all the above statistical tests will be set at *p* ≤ 0.05, indicating a significant statistical difference.

Regarding qualitative data analysis, one researcher will transcribe the interview audio recordings within 24 h after the interview and enter the transcriptions into Nvivo 12 (QSR International Pty Ltd), and another researcher will check these transcriptions. Nvivo 12 will be used for coding, and data will be analyzed and collected in parallel. Qualitative content analysis will be applied to the data collected (40). This method is an objective, systematic and quantitative research method applied to text content, which is based on the generation of explicit and descriptive content categories and the generation implicit and explanatory content themes. First, researchers will read the transcripts multiple times to get a sense of the data as a whole. Secondly, a series of open codes will be identified, and similar and related codes will be classified into subcategories. These subcategories will be then abstracted into generic categories, and finally, general categories will be summarized into main categories. Two researchers will analyze the data at the same time and compare their coding frameworks. If there is a disagreement, a third researcher will be consulted until an agreement is reached to improve the quality of the analysis.

### 2.9 Ethical approval and trial registration

The research protocol followed the SPIRIT guidelines (28), has been reviewed and approved by the clinical research ethics committee of the School of Nursing of Jilin University (number: 2024082703), and has been registered in the Chinese Clinical Trial Registry (number: ChiCTR2400094580). The confidentiality and anonymity of participant data will be assured throughout the entire process, including during the implementation of the experimental protocol, as well as for any subsequent presentations or publications stemming from the study. Prior to the study, a participant information sheet will be provided to the participants, and their written informed consent will be sought to ensure compliance with ethical standards.

## 3 Discussion

The patients with disabilities, as a typical vulnerable group within the older adults population, face multiple challenges, including physical decline and unstable health conditions. Their medical and care needs are complex, and they have a more urgent demand for specialized care (41, 42). Given the multifactorial nature of their health conditions, single-dimensional or fragmented services are often insufficient. There is a growing consensus that integrated and multidimensional interventions that simultaneously address motor, cognitive, vitality, psychological, hearing, and visual functions are essential to meeting the comprehensive needs of this population. The ICOPE-based intervention, with its person-centered, multidisciplinary, and intrinsic capacity–oriented design, provides a valuable foundation for supporting older adults with disabilities. If successful, our program could provide a practical and effective long-term care intervention for intrinsic capacity issues that could benefit disabled older adults. At the same time, exploring the experiences of old patients with disabilities in participating in this long-term care intervention can help further improve and promote the long-term care system.

The old patients with disabilities generally wish to maintain a certain level of independent living and an active lifestyle, aiming to extend their self-care abilities and reduce dependency on others (43). The decline in intrinsic capacity not only directly impacts their autonomy and well-being but also leads to a significant increase in the demand for healthcare resources (44). Research has shown a significant association between the decline in intrinsic capacity and functional impairment (15–18). As functional impairment worsens, it often increases the caregiving burden on older adults, thereby putting more pressure on healthcare and social care services (45). Therefore, promoting the maintenance of a certain level of independent living ability in older adults has become a core objective for policymakers and service providers in older adult care (46, 47). In recent years, the ICOPE has shown significant potential in optimizing long-term care services for the older adults (21, 22). This model focuses on intrinsic capacity (14), emphasizing the optimization of older adults’ functions and intrinsic capacity through more comprehensive health services to improve their overall health.

Compared to traditional single-domain health interventions, the “Long-term care intervention program based on ICOPE for old patients with disabilities in nursing homes” developed in this study, based on the ICOPE framework, targets the intrinsic capacity of older adults as the core care intervention. It aims to comprehensively improve the health and quality of life of old patients with disabilities by integrating interventions across various health domains. This program not only focuses on physical function recovery but also includes care in multiple dimensions such as exercise, cognition, nutrition, sensory, and psychological aspects, fully considering the diverse health and functional needs of disabled older adults. By constructing a systematic and collaborative service framework, this program provides comprehensive and multi-layered care services to old patients with disabilities, effectively improving care quality and alleviating the caregiving pressure on nursing homes.

However, this study will also face some challenges. First, many nursing homes face imbalanced resource and funding allocation, with some lacking the necessary equipment, technical support, and staff for the intervention (48). To address this issue, the research team plans to apply for special funding to support the project. Furthermore, the team will work to optimize the internal resource allocation of institutions and develop tailored solutions based on the specific circumstances of each facility. Second, the long-term care intervention program covers multiple health aspects, requiring caregivers to have advanced knowledge and skills. However, inconsistent training in some nursing homes limits the program’s effectiveness. To address this, we plan to develop a comprehensive training program, offer expert-led courses, and regularly assess caregivers’ performance. Thirdly, each old patients with disabilities have different health conditions and needs, and existing standardized intervention programs may struggle to address individual differences (42). To overcome this challenge, this study will develop flexible and personalized interventions, adjusting them according to each older adult’s health status and needs. Fourth, the scales used are self-reported measures, which can be subject to bias. Factors such as acute illness, changes in physical function, and stressful events can impact the accuracy of participants’ responses (49). Therefore, if conditions permit, objective indicators, such as physiological indicators, will be considered alongside self-reported measures, to reduce the bias of individual subjective feelings and improve the objectivity of the assessment. Fifth, this study may be subject to selection bias, as participants are drawn from specific nursing homes that may not fully represent the broader population of older patients with disabilities. To minimize this risk, we will seek to recruit participants from multiple institutions with diverse characteristics. Finally, the old patients with disabilities in the control group will maintain their routine life and standard care practices within the nursing home. While this mirrors real-world practice, the lack of an active or attention-matched control may overestimate the intervention’s effect due to non-specific factors like increased attention or support, and this may affect internal validity.

## 4 Conclusion

Compared to the control group, old patients with disabilities who receive the long-term care intervention program may show improvements in intrinsic capacity, specifically in areas such as motor function, cognitive function, nutrition, sensory function, and psychological function. If proven effective, the intervention program could offer a practical solution to the complex health needs of older adults with disabilities, improving their well-being and reducing caregiver and healthcare burdens. Findings from the qualitative study will further inform the refinement of the intervention, improving its effectiveness, adaptability, and user satisfaction. Clinically, this study supports the integration of person-centered, function-oriented care into long-term care practice, and encourages the adoption of the ICOPE framework as a structured model.
